# Milk Fermented with *Lactobacillus fermentum* Ameliorates Indomethacin-Induced Intestinal Inflammation: An Exploratory Study

**DOI:** 10.3390/nu11071610

**Published:** 2019-07-16

**Authors:** Lourdes Santiago-López, Adrián Hernández-Mendoza, Belinda Vallejo-Cordoba, Verónica Mata-Haro, Abraham Wall-Medrano, Aarón F. González-Córdova

**Affiliations:** 1 Laboratorio de Química y Biotecnología de Productos Lácteos, Centro de Investigación en Alimentación y Desarrollo A. C. (CIAD), Carretera Gustavo Enrique Astiazarán Rosas, No. 46 Col. La Victoria, CP, Hermosillo 83304, Sonora, Mexico; 2 Laboratorio de Microbiología e Inmunología, Centro de Investigación en Alimentación y Desarrollo A. C. (CIAD), Carretera Gustavo Enrique Astiazarán Rosas, No. 46 Col. La Victoria, CP, Hermosillo 83304, Sonora, Mexico; 3 Departamento de Ciencias Químico-Biológicas, Instituto de Ciencias Biomédicas, Universidad Autónoma de Ciudad Juárez, Anillo Envolvente del PRONAF y Estocolmo s/n, Ciudad Juárez CP, Chihuahua 32310, Mexico

**Keywords:** inflammation, fermented milk, indomethacin

## Abstract

The aim of this study was to evaluate the effect of milk fermented with *Lactobacillus fermentum* J20 (FMJ20) or J28 (FMJ28) on ameliorating indomethacin-induced inflammation. Twenty-eight male C57Bl/6 mice were divided into four experimental groups: indomethacin, indomethacin + FMJ20, indomethacin + FMJ28, and untreated (control). Groups were fed fermented milk for 15 days, followed by administration of indomethacin supplied in three sub-doses over experimental period. Body weight, and food consumption were recorded. Additionally, spleen, kidney, and liver were weighed, and the small intestine length was measured. The cytokines in serum (IL-2, IL-4, IL-6, IL-10, IL-17, IL-23 and TNFα) and in intestinal mucosa (IL-17 and IFNγ) were also determined. Compared to the control, all indomethacin-supplemented groups lost weight (~2.7 g; *p* < 0.05), but no changes were found in the organ-specific morphometry analysis. FMJ28 showed better results in attenuating serum and intestinal IL-17 levels. Furthermore, showed less epithelial cell loss and inflammatory infiltrates than the other indomethacin-treated groups. These results suggest that FMJ28 may be effective in reducing intestinal and systemic acute inflammation, specifically in mice.

## 1. Introduction

Non-steroidal anti-inflammatory drugs (NSAIDs) such as indomethacin (PubChem CID: 3715) are typically used to treat chronic inflammatory diseases such as gout-like and psoriatic arthritis. However, their continuous use often causes liver disease or gastrointestinal lesions characterized by ulcerations, bleeding, and abnormal intestinal permeability [1]. From a molecular standpoint, NSAIDS can destroy epithelial tight junctions, reduce the mucus layer, promote the abnormal passage of luminal antigens and intestinal bacteria, and increase the local/systemic inflammatory response [2,3]. Several alternatives to prevent or reduce the side effects of NSAIDs include other over-the-counter drugs (e.g., cyclooxygenase-2 selective agents, proton pump inhibitors) and probiotic bacteria [4,5]. However, the molecular pathogenesis of NSAID-related inflammatory lesions is not fully understood and, currently, there are no certified clinical strategies for preventing NSAID-induced small intestinal injury.

The proper balance of pro- and anti-inflammatory cytokines is necessary to preserve intestinal integrity. TNFα is a pro-inflammatory cytokine produced by activated monocytes/macrophages [6,7], so one possible additional strategy to mitigate inflammation is the use of anti-TNFα monoclonal antibodies. However, the persistent and marked blockage of TNFα bioactivity may have a detrimental effect on acute intestinal inflammation [8]. TNFα is involved in the early stages of NSAID-induced intestinal inflammation and up-regulates the presence of IL-1β and inducible nitric oxide synthase (NOS) [9]. IL-17 is another pro-inflammatory cytokine produced by T-cells (CD4^+^ TCRαβ and CD8+αα TCRγδ) within *lamina propria*; however, its involvement in the inflammatory process within the small and large intestines is not entirely clear. Some studies suggest the role of IL-17A in the process small intestinal injury induced by indomethacin, as well as in the up-regulation of granulocyte-colony stimulating factor (G-CSF), keratinocyte chemoattractant (KC), and monocyte chemotactic protein-1 (MCP-1) [10,11].

Considering that probiotic bacteria have shown to exert a protective effect on Lipopolysaccharide (LPS)-induced colon inflammation [12,13], some few studies examining the potential effect of probiotic bacteria on indomethacin-induced small bowel enteropathy have also been carried out. In this sense, it has been reported that viable cells of *Lactobacillus casei* Shirota prevented indomethacin-induced intestinal injury by decreasing pro-inflammatory markers (e.g., TNFα), adhesion molecules (e.g., KC, MCP-1), myeloperoxidase activity, and the iNOS mRNA level in addition to up-regulating the TLR4 mRNA level [5]. On the other hand, contradictory results have been found for other lactic acid bacteria. For instance, Kamil et al. [14] reported that *Lactobacillus rhamnosus* GG (LGG) did not alleviate indomethacin-induced intestinal injury in rats with enteropathy; in contrast, in other study LGG did protect the integrity of the gastric mucosal barrier (but not the intestinal mucosal barrier) against indomethacin in humans [15]. Furthermore, in a double-blind crossover trial, the administration of a probiotic mixture (VSL#3: *Streptococcus thermophilus*, *Bifidobacterium longum*, *Bifidobacterium breve*, *Bifidobacterium infantis*, *Lactobacillus acidophilus*, *Lactobacillus plantarum*, *Lactobacillus casei*, and *Lactobacillus bulgaricus*) reduced fecal calprotectin, a neutrophil-specific biomarker of intestinal inflammation [16]. All these results indicate that direct administration of probiotics bacteria may prevent, in a strain-specific manner, the damage induced by indomethacin. Additionally, the effectiveness of an immune-modulating diet, comprising whey peptides and non-probiotic fermented milk as protein source, on indomethacin-induced small-bowel disorders was determined. Results showed that immune-modulating diet significantly reduced increased permeability of the mucosa, bacterial translocation in the mesenteric lymph nodes, and concentrations of IL-6 in the ileal tissues compared with the control group [17]. These results evidenced the potential immunomodulatory effects of hydrolysates and peptide fractions derived from milk proteins on indomethacin-induced small-bowel disorders. However, to the best of our knowledge, there are no studies addressing the effect of probiotic-rich foods, such as fermented dairy products. Hence, the aim of this study was to evaluate the effect of milk fermented with potential probiotic *Lactobacillus fermentum* J20 (FMJ20) or J28 (FMJ28) on ameliorating indomethacin-induced inflammation.

## 2. Methods

### 2.1. Preparation of Fermented Milk

The strains *Lactobacillus fermentum* J20 and J28, previously isolated from Mexican artisanal cheese, were selected on the basis that these bacteria have already exhibited both technological and probiotic potential [18], conjugated linoleic acid [19], and anti-inflammatory and immunomodulatory properties, by using models of colon inflammation with LPS [20] or Dextran Sulfate Sodium (DSS) [21], and healthy Wistar rats [18], respectively. Bacteria were grown in MRS (De Man, Rogosa, and Sharpe, Difco) broth (12 h, 37 °C). Previously, commercial skimmed milk powder was reconstituted (10% *w*/*v*) in water and sterilized (110 °C, 10 min). Then, milk was inoculated (3% *v*/*v*) with fresh culture (12 h) of each bacterium (*Lactobacillus fermentum* J20 or J28) and incubated during 48 h at 37 °C. Finally, the fermented milks (FMs) were placed in a cold water bath until use. At the end of fermentation, the cell concentration was evaluated by plate count on MRS agar plates incubated for 48 h at 37 °C, and the results were reported as colony forming units (CFU/mL). The protein content was evaluated by the Kjeldahl method (939.02 AOAC International, 2006) [22], and lactic acid (947.05 AOAC International, 1990) [23], as well as, pH (HI 2211 pH and ORP Benchtop Meter, Hanna Instruments, Woonocket, RI, USA) were also determined.

### 2.2. Animal Study and Induction of Inflammation

Twenty-eight C57Bl/6 mice (weight 30.46 ± 5.41 g, six weeks old) were obtained from BIOINVERT (Mexico City, Mexico). Mice were housed in a controlled environment (22 °C, 12 h/12 h light/dark cycle). Acute inflammation was induced by the administration of indomethacin (Sigma-Aldrich, Mexico) at a concentration of 10 mg/kg of body weight [11].

The mice were randomly separated into four groups (*n* = 7): (a) untreated control (control), (b) indomethacin (IND), (c) *Lactobacillus fermentum* J20 (FMJ20) + IND, and (d) *Lactobacillus fermentum* J28 (FMJ28) + IND. The weight of each animal was taken as a variable factor for randomized, and randomly assigned numbers were generated (Microsoft Excel, Redmond, WA, USA, 2016). Hence, the means of the weight were compared by a one-way ANOVA. The groups were formed to obtain a probability level of *p* = 0.09. The animals of each group had an initial weight of 33.28 ± 7.01, 33.22 ± 5.59, 38.14 ± 4.56, and 36.52 ± 6.15 (*p* > 0.05), respectively.

**Figure 1:**
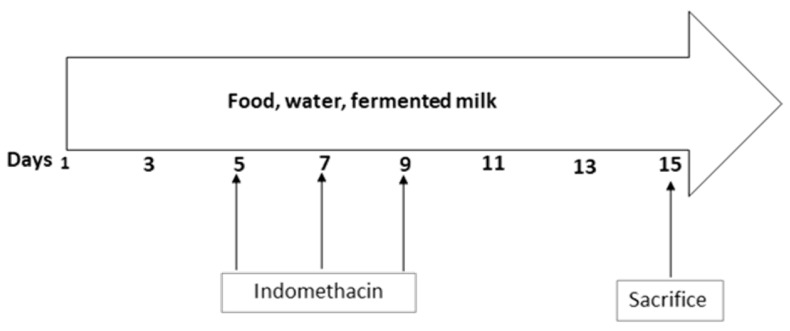
Administration of indomethacin and fermented milk. Food and water were provided ad libitum.

The mice were fed a normal diet (2018 Teklad Global, 18% protein rodent diet, ENVIGO) and water ad libitum. Food and water consumption and the weight of mice were recorded daily. The mice were administered by oral gavage 1 mL/day of FMJ20 or FMJ28 with a cell concentration of 9 × 10^8^ and 1 × 10^9^ CFU/mL, respectively, and three doses of indomethacin during the experimental trial (day 5, 7, and 9) (Figure 1). The study was approved by the Bioethics Committee of the Research Center for Food and Development (CIAD A.C.), Hermosillo, Sonora, Mexico (CE/002/2015).

### 2.3. Biological Samples

At the end of the experiment (15 days), the mice were sacrificed, and the spleen, kidney, liver, and small intestinal were obtained. The organs were weighed, and the length of the small intestine was measured. Blood samples were collected by cardiac puncture and maintained at room temperature for 30 min. Subsequently, the serum was harvested by centrifugation (3500 rpm, 5 min, 4 °C) and stored at −80 °C until further cytokine analysis.

The ileum section was cut longitudinally and resuspended in 1 mL of RPMI 1640 medium (Sigma-Aldrich, Mexico) supplemented with 1% penicillin-streptomycin and 10% fetal bovine serum. The samples were incubated at 37 °C for 24 h. The supernatants were harvested by centrifugation (3600× *g*, 10 min, 10 °C) and stored at −80 °C for cytokine analysis [24].

### 2.4. Cytokines

Serum samples and supernatants of the small intestine were used for the quantification of cytokines. Specifically, ELISA kits (R & D Systems, San Jose, CA, USA) were used to determine IFNγ, IL-6, IL-17, and IL-23 following the manufacturer’s instructions. In addition, IL-2 (detectable minimal dose [DMD] 0.1 pg/mL), IL-4 (DMD 0.03 pg/mL), IL-10 (DMD 6.8 pg/mL), and TNFα (DMD 0.9 pg/mL) (Becton Dickinson and Company, San Jose, CA, USA) were evaluated by flow cytometry (BD FACSCanto^TM^ II, San Jose, CA, USA) and date were analyzed used the FCAP array Software (BD San Jose, CA, USA). These assays provided a method of capturing soluble analytes or a set of analytes with beads of known size and fluorescence.

### 2.5. Histological Analysis

A small portion (0.5 cm) of each intestinal sample was fixed in 10% buffered formalin for at least 72 h, dehydrated in ethanol, embedded in paraffin, and sliced. Samples were then deparaffinized, rehydrated, and stained with hematoxylin and eosin staining. The samples were evaluated under a 20× light fluorescent microscope (Carl Zeiss, Jena, Germany), and the morphological changes in the tissues were observed. The images were processed in the Zeiss AxionVision 4.8.3 software using a 20× objective (NA 1.3, PlanNeoFluar, Carl Zeiss, Göttingen, Germany). Histological examinations were performed in triplicate as previously described [21].

### 2.6. Statistical Analysis

Data were expressed as means ± SDs. Body weight and organ morphometric data from all experimental groups were analyzed by one-way ANOVAs and compared using post hoc Tukey-Kramer tests (*p* < 0.05). Plasma and intestinal cytokine values were analyzed using non-parametric tests, and the data were analyzed by the Kruskal-Wallis test. Differences were considered statistically significant when *p* < 0.05. All statistical differences were carried out in analyzed the Minitab V.18.1.1. software.

## 3. Results and Discussion

FMJ20 and FMJ28 (48 h) showed a cell concentration of 9 × 10^8^ ± 1 × 10^7^ and 1 × 10^9^ ± 2 × 10^7^ CFU/mL, total protein content of 3.01 ± 0.15% and 2.99 ± 0.04%, lactic acid content of 0.84 ± 0.01% and 0.87 ± 0.03%, and pH of 4.18 ± 0.03 and 4.05 ± 0.01, respectively. Statistical differences (*p* < 0.05) were not found between the milks. Bacterial cell concentrations established in fermented milks have shown immunomodulatory and anti-inflammatory effects in previous works [5,14,15,16,18,21], which suggests that bacterial populations found here may be effective on ameliorating induced inflammation.

Compared to healthy animals (controls), all indomethacin-supplemented groups lost weight (~2.7 g; *p* < 0.05) (Table 1). The weight loss was particularly pronounced after the second dose of indomethacin, at which point the body weight of mice in the experimental groups significantly decreased compared to the control group. The loss of weight was mainly attributed to the administration of indomethacin because no significant differences were found in food consumption among groups during the experimental trial (Table 2). After the second dose of indomethacin, two mice died in the IND and FMJ20 groups. As a result, these two groups were reduced to five individuals, and the final analyses only contemplated these individuals.

The kidney and liver samples did not differ in weight among treatment groups. However, the spleen weight increased for the IND group (288 ± 74.08 mg) (*p* > 0.05), similar to the FMJ20 group. The FMJ28 group only showed a minor gain with respect to the IND and FMJ20 groups.

The small intestine length of mice in the FM groups did not significantly differ between indomethacin and control groups (*p* > 0.05), although the small intestine length of the indomethacin group decreased with respect to the control group. In this regard, Yamada et al. [11] reported that a 24-h induction of inflammation with a single dose (10 mg/kg) of indomethacin results in multiple erosions and ulcers in the small intestine, affecting intestine length. In fact, the authors reported that intestinal injuries are detectable as soon as three hours after the administered dose. However, this was not the case in this study, which were three doses of indomethacin over 15 days versus a single dose followed by sacrifice used by Yamada et al. [11].

**Figure 2:**
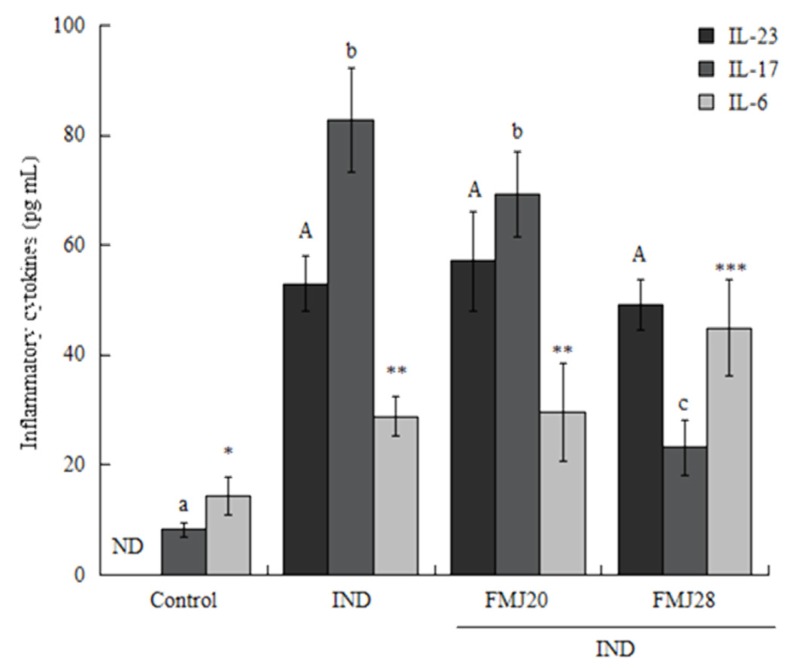
Effect of administration of fermented milk on the concentration of inflammatory cytokines in serum samples of mice with indomethacin-induced inflammation determined by ELISA assay. The values represent the medians + interquartile ranges of n = 7 for the control and FMJ28 groups and n = 5 for the IND and FMJ20 groups. Capital letters show statistical differences for IL-23, lowercase letters for IL-17, and *, and *** show statistical difference, and ** asterisks, the samples are same for IL-6. The Kruskal-Wallis test was used to compare values (*p* < 0.05). IND = Indomethacin only, FMJ20 = Fermented milk J20, FMJ28 = Fermented milk J28, ND = Not detected.

Figure 2 shows the results obtained for cytokines in serum. The indomethacin-only group showed significantly (*p* < 0.05) higher levels of IL-17 with respect to the control and FMJ28 groups. IL-6 was only significantly higher (*p* < 0.05) in the FMJ28 group compared to the control, IND, and FMJ20 groups.

IL-23 did not differ statistically among treatments and was not detected in the control group. This cytokine has been related with the regulation of mucosal inflammation at the gastrointestinal level. In patients with inflammatory bowel disease, the concentration of IL-23 is elevated. This cytokine also promotes the growth of colon cancer. Therefore, the suppression of IL-23 and its receptors might decrease the inflammatory process, as previously reported [25]. In fact, the presence of this cytokine is due to the activation of antigen-presenting cells such as macrophages and dendritic cells [26]. Under the experimental conditions of our study, the bacteria and metabolites present in the fermented milk could be activating these cells through binding with toll-like receptors, leading to the activation of the transcription factor retinoic acid [27], which was previously suggested by some studies to occur in the presence of probiotic bacteria.

**Figure 3:**
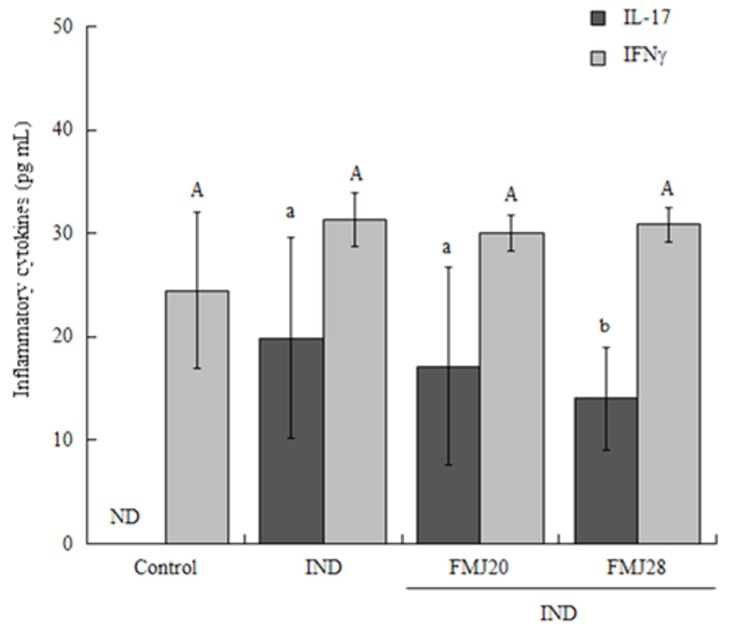
Effect of the administration of fermented milk on the concentration of inflammatory cytokines in small intestinal mucosa samples of mice with indomethacin-induced inflammation determined by ELISA assay. The values represent the medians + interquartile ranges of *n* = 7 for control and FMJ28 and *n* = 5 for IND and FMJ20. Capital letters show the statistical differences for IL-17 and lowercase letters for IFNγ. Kruskal-Wallis test was used to compare values (*p* < 0.05). IND = Indomethacin only, FMJ20 = Fermented milk J20, FMJ28 = Fermented milk J28, ND = Not detected.

On the other hand, in the intestinal samples, IFNγ was enhanced in all treatment groups (*p* > 0.05) (Figure 3). The concentration of IL-17 did not differ statistically among treatment groups except for the FMJ28 treatment; in the control, it was not detected.

Conversely, the cytokines IL-2, IL-4, IL-10, and TNFα did not show significant differences in the serum samples (Table 3). These results may indicate that any major effects might occur at the local level, as some studies have suggested that specific cytokines play a role in mucosal immunity [28]. The immune system associated with the intestinal mucosa and the presence of cytokines are important for maintaining normal gastrointestinal homeostasis, but an imbalance can favor inflammatory processes. Meanwhile, anti-inflammatory cytokines, such as IL-10 and IL-4, contribute to decreasing the inflammatory response in the intestinal mucosa [29]. In a previous study, the administration of fermented milk with J20 and J28 showed the capacity to regulate the cytokines IL-6 and IL-10 in serum samples after 21 days in a healthy murine model [18]. Although the mechanisms by which these bacteria activate the immune response have not been evaluated, other studies have suggested, for example, that the administration of *Bifidobacterium breve* in mice interacts with dendritic cells, bind to TLR2, a recognized polysaccharide structure of the cell wall, promote Trl cells, and induce Foxp3+ [30]. It is possible that the strains utilized in our study or their metabolites are having similar interactions.

Several authors maintain that IL-17 plays a key role in the inflammatory process through regulating other pro-inflammatory cytokines and chemoattractant factors [7,31]. Yamada et al. [11] reported that the mRNA levels of IL-17A in the small intestine increased after the administration of indomethacin. Conversely, the expression of KC, MCP-1, and G-CSF involved in chemotaxis and MPO activity was suppressed in IL-17^−/−^ mice. In our preceding study [21], we reported increased serum levels of IL-17 in mice with DSS-induced inflammation. Also, FMJ28 led to the higher production of bacterial exopolysaccharides, possibly related with its ameliorating effect on IL-17 levels.

**Figure 4:**
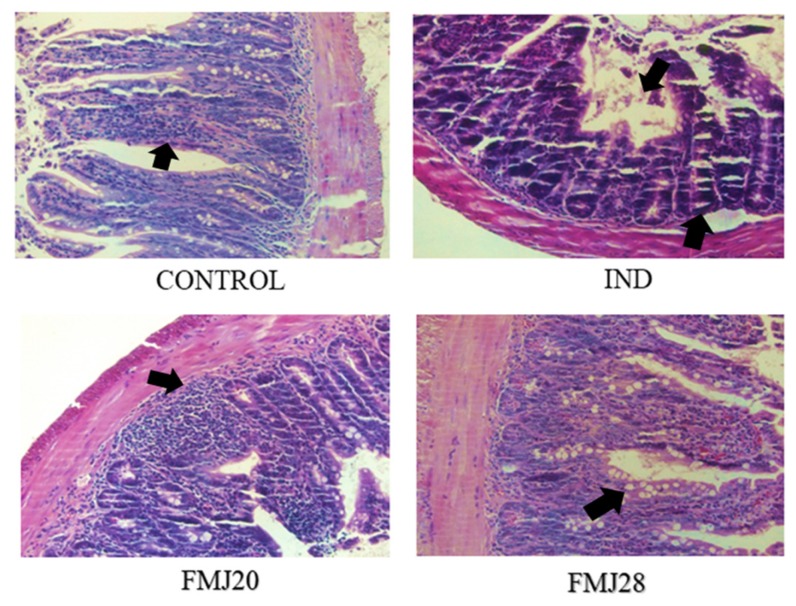
Histological findings in the small intestine after indomethacin administration. All images were taken using a 20× objective. IND = Indomethacin only, FMJ20 = Fermented milk J20, FMJ28 = Fermented milk J28. Black arrow show effect on the epithelial structure by IND and FM.

The histological samples of the small intestine of the treatment groups stained with hematoxylin-eosin (20×) showed intestinal lesions, the destruction of the epithelium, and the infiltration of inflammatory cells in the submucosa compared to the control mice (Figure 4). Less severe injury, milder inflammation, and minor cell infiltration and injury were observed in epithelial cells administered with FMJ28. Also, regular crypts were observed compared to the IND group. In this group, the crypt abscess formation. Other studies have demonstrated that cytokines such as TNFα promote indomethacin-induced injury in mice as a result of the acute inflammatory response accompanied with neutrophil accumulation and the up-regulation of KC chemokine [6]. Our results are similar to those reported by Harusato et al. [32], in which the administration of indomethacin resulted in defects in the villi, epithelial stratification, basal lamina degeneration, and cell infiltration.

The enhancement of inflammation following indomethacin administration may be attributed to the inhibition of cyclooxygenase and prostaglandin, which are mediators in the inflammatory response that maintain the homeostasis of many tissues and organs [33]. Mucosal injuries can also be caused by the penetration of bile acid, proteolytic enzymes, and intestinal bacteria or toxins and the activation of receptors such as TLR4 [34,35]. The inhibition of oxidative phosphorylation in the mitochondria may be the main activation mechanism due to the inhibition of phosphorylation and the increase in the permeability to intestinal level. This mechanism is possibly a result of ATP deficiency following the inhibition of oxidative phosphorylation. Also, the leakage of calcium in the mitochondria causes an increase in cytosolic Ca^2+^. Reactive oxygen species may then increase and modify the Na^+^/K^+^ channels, consequently affecting the tight intracellular junctions and enhancing intestinal permeability [36].

Our results indicate that the administration of FMJ28 prior to the administration of indomethacin enabled protection from inflammation at the intestinal level, possibly as a result of the adherence of *Lactobacillus fermentum* to epithelial cells or EPS-production during the fermentation process [21]. Also, the presence of lactic acid may have had an anti-inflammatory effect, as previously reported [5,21]. Our study did not explore the possible mechanisms of action of bacteria or their metabolites, yet a prior study suggested that the action of VSL#3 reduced intestinal permeability, modulating the tight junction proteins, occludin, and claudin-2 [37]. It is possible that the bacteria in our study can also up-regulated the inflammatory cytokines, as well as, inhibit intestinal permeability.

Additional studies have shown that probiotic bacteria may have positive effects on inflammatory bowel disease and irritable bowel syndrome. For example, the administration of *Lactobacillus casei* Shirota improved the inflammatory process in LPS-stimulated large intestinal lamina propria mononuclear cells and in vivo models induced by dextran sodium sulfate [38]. Specifically, the administration of this probiotic for one week prevented indomethacin-induced intestinal injury, inhibited increases in myeloperoxidase activity and TNF-α mRNA expression, and also affected TLR4 expression; however, heat-killed or single doses of viable cells did not inhibit intestinal injury [5]. Kamil et al. [14] reported that the administration of *Lactobacillus rhamnosus* GG (LGG) increased the MPO activity by 2.3-fold to 9.8-fold in the ulceration area compared to an indomethacin-only treatment group (*p* > 0.05). It appears in this latter case that the administration of LGG and Bb12 enhanced the inflammatory response. However, this effect may be attributed to the small increase in permeability and the reduction in microcirculation and prostaglandin production induced by indomethacin [33]. Another study in humans showed that the regular consumption of live LGG may protect the integrity of the gastric mucosal barrier against indomethacin but has no effect on intestinal permeability [15].

Notably, a period of probiotic pre-treatment seems to be necessary to prevent intestinal injury. In a double-blind, crossover trial, 20 healthy volunteers consumed a dose of probiotics (VSL#3) or a placebo treatment (corn starch) for 21 days in addition to 50 mg/day of indomethacin. The fecal calprotectin concentrations significantly increased only at day 17 with respect to the initial concentrations prior to the experiment, whereas these concentrations continually increased from days 10 to 21 in those undergoing the placebo treatment [16]. Specifically, calprotectin is a calcium-binding protein found in neutrophilic granulocytes, monocytes, and macrophages that resists metabolic degradation. It can be measured in feces and used as an indicator of the response and number of neutrophils during the inflammation process [39].

Overall, the results inflammatory in the present study showed that the combination of *Lactobacillus fermentum* strains and metabolites released during the fermentation decreased the effect of indomethacin-induced inflammation in the small intestine of mice. This corroborates the findings of in vivo and clinical studies that have demonstrated the potential of probiotic administration or consumption for decreasing the inflammatory response induced by indomethacin. However, uncertainties remain regarding the most adequate probiotic strains, the correct dose, and the efficacy of probiotic mixtures versus single strains. Although the administration of FM may inhibit the inflammatory process by regulating inflammatory cytokines in serum and intestinal mucosa, the mechanisms underlying indomethacin-induced neutrophil migration and inflammatory cytokine expression in the small intestine are unclear. It is important to further evaluate the effect of viable cells because probiotic colonization may be necessary for a probiotic effect to be exerted in the small intestine.

## 4. Conclusions

In the current exploratory study, the administration of indomethacin showed a significant reduction of body weight and histological intestinal lesions. Furthermore, significant higher levels of proinflammatory cytokines (i.e., IL-6, IL-17, IL-23 and IFNγ) were observed, either systemically or locally, in all indomethacin-treated groups. These data evidenced an induced ileal inflammation. In contrast, both histological intestinal lesions and high levels of IL-17 induced by indomethacin, were reduced by the administration of fermented milk, particularly by fermented milk with *Lactobacillus fermentum* J28 (FMJ28). Hence, our findings suggested that FMJ28 may be effective in reducing intestinal and systemic acute inflammation, specifically in mice. However, further studies are needed to elucidate the possible bioactive components of fermented milk and their potential mechanisms underlaying the anti-inflammatory effect.

## Figures and Tables

**Table 1:** Effect of fermented milk treatments on organ weight and small intestine length.

Group	Weight	Weight (mg)			Length (cm)
	Loss (g)	Spleen	Kidney	Liver	Intestine
Control	−3.7 ± 3.4 ^a^	140 ± 97.6 ^a^	453 ± 92.2 ^a^	1580 ± 313.8 ^a^	50.5 ± 4.5 ^a^
IND	−5.8 ± 3.7 ^b^	288 ± 74.1 ^a^	500 ± 352.4 ^a^	1764 ± 1045.1 ^a^	45.9 ± 8.0 ^a^
FMJ20	−6.3 ± 2.8 ^b,c^	294 ± 74.0 ^a^	652 ± 99.9 ^a^	2318 ± 363.0 ^a^	45.0 ± 4.6 ^a^
FMJ28	−6.9 ± 3.6 ^b,c^	116± 131.8 ^a^	545 ± 366.7 ^a^	1274 ± 1146.0 ^a^	43.6 ± 2.0 ^a^

Results shown as means ± SD. Different letters per column indicate statistical differences among experimental groups (*p* < 0.05). IND = Indomethacin only, FMJ20 = Fermented milk J20, FMJ28 = Fermented milk J28.

**Table 2:** Monitoring of feed intake during the experimental trial.

Group	Food Consumption (g)						
	Days						
	1	3	5	7	9	12	14
Control	7.24 ± 2.23 ^a^	8.08 ±1.25 ^a^	6.28 ± 2.15 ^a^	7.03 ±1.75 ^a^	6.15 ± 2.11 ^a^	6.17 ± 1.45 ^a^	6.23 ± 1.89 ^a^
IND	8.24 ± 1.34 ^a^	7.08 ± 1.25 ^a^	7.28 ± 2.45 ^a^	6.03 ± 2.34 ^b^	5.15 ± 1.67 ^b^	5.17 ± 1.56 ^b^	5.23 ± 1.23 ^b^
FMJ20	6.95 ± 1.45 ^a^	7.15 ± 1.78 ^a^	7.38 ± 1.23 ^a^	5.92 ± 1.67 ^c^	6.02 ± 1.98 ^c^	7.18 ± 1.27 ^a^	6.01 ± 1.38 ^a^
FMJ28	8.13 ± 1.98 ^a^	8.71 ± 1.56 ^a^	6.47 ± 1.49 ^a^	6.97 ± 1.78 ^b^	6.08 ± 1.23 ^c^	6.86 ± 1.45 ^a^	7.23 ± 2.22 ^a^

Results are shown as means ± SDs. Different letters per column indicate statistical differences among experimental groups (*p* < 0.05). IND = Indomethacin only.

**Table 3:** Effect of fermented milk treatments on the regulation of inflammatory cytokines in serum samples.

Cytokines	Concentration (pg/mL)			
	Control	IND	FMJ20	FMJ28
**IL-2**	6.78 ^a^	4.33 ^a^	4.76 ^a^	5.18 ^a^
**IL-4**	3.60 ^a^	5.83 ^a^	5.67 ^a^	5.49 ^a^
**IL-10**	ND	ND	ND	ND
**TNFα**	6.33 ^a^	3.47 ^a^	3.38 ^a^	4.37 ^a^

Results are shown as medians. Different letters per column indicate statistical differences among groups (*p* < 0.05). IND = Indomethacin only, FMJ20 = Fermented milk J20, FMJ28 = Fermented milk J28, ND = Not detected.
